# What People Want to Know About Their Genes: A Critical Review of the Literature on Large-Scale Genome Sequencing Studies

**DOI:** 10.3390/healthcare6030096

**Published:** 2018-08-08

**Authors:** Courtney L. Scherr, Sharon Aufox, Amy A. Ross, Sanjana Ramesh, Catherine A. Wicklund, Maureen Smith

**Affiliations:** 1 Center for Communication and Health, Department of Communication Studies, Northwestern University, 710 North Lake Shore Drive, 15th Floor, Chicago, IL 60611; USA; amyross2019@u.northwestern.edu (A.A.R.); sanjanaramesh2022@u.northwestern.edu (S.R.); 2 Center for Genetic Medicine, Feinberg School of Medicine, Northwestern University, 645 N Michigan Ave, Suite 630, Chicago, IL 60611, USA; s-aufox@northwestern.edu (S.A.); c-wicklund@northwestern.edu (C.A.W.); m-smith6@northwestern.edu (M.S.)

**Keywords:** all of us, genetic studies, participant expectations, precision medicine, public health, results return

## Abstract

From a public health perspective, the “All of Us” study provides an opportunity to isolate targeted and cost-effective prevention and early-detection strategies. Identifying motivations for participation in large-scale genomic sequencing (LSGS) studies, and motivations and preferences to receive results will help determine effective strategies for “All of Us” study implementation. This paper offers a critical review of the literature regarding LSGS for adult onset hereditary conditions where results could indicate an increased risk to develop disease. The purpose of this review is to synthesize studies which explored peoples’ motivations for participating in LSGS studies, and their desire to receive different types of genetic results. Participants were primarily motivated by altruism, desire to know more about their health, and curiosity. When asked about hypothetically receiving results, most participants in hypothetical studies wanted all results except those which were uncertain (i.e., a variant of uncertain significance (VUS)). However, participants in studies where results were returned preferred to receive only results for which an intervention was available, but also wanted VUS. Concerns about peoples’ understanding of results and possible psychosocial implications are noted. Most studies examined populations classified as “early adopters,” therefore, additional research on motivations and expectations among the general public, minority, and underserved populations is needed.

## 1. Introduction

Over three years ago, President Obama announced the Precision Medicine Initiative (PMI), a research effort to personalize medicine based on an individual’s genes, environment, and lifestyle [1]. Since the PMI announcement, research has intensified to improve the understanding of disease mechanisms, diagnosis, and treatment outcomes using genomic sequencing projects [2]. From a public health perspective, the promise of the PMI is the identification of personalized interventions that not only include treatment, but also more targeted and cost-effective prevention and early-detection strategies [3,4,5]. Furthermore, hope exists that providing people with individualized genetic risk information will inform prevention and early-detection behaviors [6]. Yet, these outcomes will not be achieved without efforts to identify effective strategies for study implementation that ensure generalizability and motivate prevention behaviors. 

Prior to the PMI, several federally funded research consortia were established to ascertain best practices in genomic sequencing research and practice. These consortia examined practical challenges related to recruitment and informed consent, data management, big data analysis, information and data sharing, and the dissemination and use of genetic results [7]. The PMI aims to use knowledge gained from these consortia to create a nationally-representative database with one million individual genetic profiles, called the “All of Us Research Program”, led by the National Institutes of Health (NIH). Recruitment is underway, and plans to return genetic results are in development (see: https://allofus.nih.gov/), which makes the present an ideal time to consider how efforts from consortia studies can inform practices and future research efforts surrounding the PMI to ensure the advancement of public health goals.

This paper offers a critical review of existing literature related to participation in hypothetical and actual genomic research in the United States. The purpose of a critical review is not to aggregate all existing literature on the topic under review, but rather to extract what is valuable to inform conceptual development [8]. As such, we reviewed existing literature to identify, analyze, and synthesize studies exploring expectations and motivations for participating in LSGS studies, and participants’ desires to receive different types of genetic results. The purpose of this methodology is to organize competing attitudes towards reasons for participation in LSGS studies, and preferences towards receiving results [8]. In doing so, we hope to synthesize what is currently known to inform recruitment processes and guideline development for the return of results. We conclude by highlighting gaps in existing literature and provide suggestions for future investigation.

## 2. Methodology

Given our emphasis on population health, we explored studies focused on large-scale genomic sequencing (LSGS) for adult onset hereditary conditions where results could indicate an increased risk to develop disease, and excluded LSGS testing for clinical purposes, for conditions in which disease onset is certain (e.g., Huntington’s), or for pediatric conditions. We use the acronym LSGS to refer to testing where many genes are being analyzed at once, including genome-wide association studies (GWAS), whole exome and/or whole-genome sequencing (WES/WGS), and/or targeted sequencing of a large panel of genes. In addition, because guidelines, cultural, and social norms vary by country with respect to genetic testing, and genetics in general, we focused on studies carried out in the United States. 

We conducted an initial search between 2015 and 2016 by examining the websites of existing LGSG studies which had a listing of publications. For example, Clinical Sequencing Evidence-Generating Research (CSER): https://cser-consortium.org/publications, Electronic Medical Records and Genomics (eMERGE): https://emerge.mc.vanderbilt.edu/publications/, and CLINSEQ: https://www.genome.gov/25521307/clinseq-study-news--updates/. A study team member reviewed the listing of publications for those which described participation in LSGS studies. In addition to selecting relevant manuscripts from the publication list, we entered manuscript titles into the Web of Science to identify additional relevant papers cited by or citing the paper from the publication listing. We also used Web of Science, Google Scholar, and our institution’s library using search terms including patient and “experiences”, or “expectations”, or “motivations” plus “genetic testing”, “GWAS”, or “WGS”, or “WES”. At the beginning of 2018, two study team members updated the list of publications by entering in each earlier identified manuscript to the Web of Science search to determine any literature that had cited the previous studies since 2015. Likewise, the websites and publication listings of relevant LSGS studies were reviewed from 2015 to the present for any additional relevant publications (see Table 1 for the list of included publications). 

## 3. Motivations for Participation in LSGS Studies

The success of the “All of Us Research Program” and other similar studies examining links between genetics and health outcomes depend on the enrollment of large numbers of people willing to longitudinally share their data with researchers [20,21]. Some studies examined hypothetical participation on LSGS studies. Other studies are “proof-of-principle” studies, designed to accumulate evidence about the impact of introducing new genomic sequencing technologies in defined populations [9,22]. These studies have evolved over time, some began by only recruiting populations with disease, or those who were at risk of developing disease, and later included healthy individuals (e.g., ClinSeq). We first discuss motivations for participating in LSGS studies and then explore preferences for the return of results from LSGS studies. 

### 3.1. Intrinsic Motivations for Participating in LSGS Studies

Exploring peoples’ motivations to enroll in LSGS studies is an important aspect of federally funded LSGS research consortia efforts. Most studies found participants held multiple motivations for participating in LSGS studies, but three motivations were consistent across studies: altruism, personal/family benefit, and curiosity. 

### 3.2. Altruism

Debates regarding the definition of altruism are beyond the scope of this paper see: [23,24,25]. Given existing debates, it is unsurprising that studies used the term altruism inconsistently, if at all. When the term altruism was used, slight variations in operational definitions emerged. For the purposes of this review, and consistent with prior definitions of altruism in research studies, we define altruism as a prosocial behavior including either or both aspects: (1) the desire to help others; and (2) the desire to advance research or science. When authors did not use the term altruism, we still categorized it as such, based on the aforementioned definition.

Interviews conducted with a subsample of unselected members of the general population in the longitudinal HealthSeq study identified altruism as a motivator, which was framed as either “an altruistic desire to help others and the field of medicine”, or “to contribute to the advancement of science” [10]. Altruism in the ClinSeq study was operationalized as a desire to help someone who may be at risk of specific disorders, such as coronary artery disease (CAD), to “help others in the future”, and advance research in genetics or health [9]. However, the survey conducted with unselected volunteers in the Coriell Personalized Medicine Collaborative (CPMC) only included the response option, “participating in research to help others”, and did not include a response option regarding the opportunity to advance science [11]. Of note, the authors did not describe this as altruism. In contrast, responses from the NextGen study focused on the advancement of research, but not on helping others, and again, the authors did not refer to this type of response as altruism [12]. 

Altruism was identified across studies as motivating participation in LSGS, but the number of people who reported altruism as a motivating factor varied. In qualitative interviews conducted prior to genetic results disclosure in the ClinSeq study of affected or at-risk participants, altruism was found to be a motivator for approximately half of participants. For example, approximately 44% of participants with or at risk of CAD reported altruism as a primary motivation for taking part [9]. Similarly, altruism was a motivating factor for unselected (i.e., healthy) participants. The CPMC, a study in which unselected volunteer participants receive ‘medically actionable’ genetic test results, found 56.2% of participants indicated “participating in research to help others” was a “very important” reason for taking part in the study [11]. 

The most divergent finding was in the NextGen study, which explored motivation to participate in LSGS research among healthy women and couples actively planning a pregnancy [12]. Participants were asked an open-ended question after enrollment about “what they hoped to learn from being in the study.” Compared with the aforementioned studies, support of research was the least reported motivation (11%); however, the lower rate could be influenced by the way the question was asked. 

### 3.3. Personal and Family Benefits

Interestingly, responses from the ClinSeq cohort, indicated motivation for participation was either driven by altruism or seeking personal health information, with little overlap [9]. Those who reported seeking personal health information wanted to learn about their risk for CAD, while others wanted information about genetic risk and predispositions to disease more generally, especially in the case of a family history of disease [9]. Most other studies found motivations for participation often included both altruism and a desire for personal information, among other motivators. In contrast with LSGS studies of affected or at-risk populations, studies of unselected or healthy participants more often cited personal benefit as the primary motivator for participation [10,11,12]. For example, in qualitative interviews conducted with unselected individuals, Sanderson, Linderman, Suckiel, Diaz, Zinberg, Ferryman, Wasserstein, Kasarskis and Schadt [10] found most participants reported the potential benefit for themselves and their family, some of whom also indicated altruism. Related to personal benefit, participants were hopeful that information from genetic testing could help them avoid or reduce their risk of disease, or help them to prepare or plan for the future. In some cases, they did not expect information from genetic testing would be immediately available or beneficial, but hoped additional research and investigation would provide more information in the future. 

Among unselected healthy participants in the CPMC, approximately 78% indicated finding out about diseases for which they were at risk, and finding out what they can do to improve their health as very important motivators for participation [11]. Half of participants were interested in risk information related specifically to either heart disease, diabetes, or cancer. Of note, a minority (3.3%) wanted to know their risk of Alzheimer’s disease, even though they were told at enrollment this information would not be available. A smaller proportion (47%) reported obtaining information about risk of health conditions for children and grandchildren as a motivating their participation. Consistently, the NextGen study of healthy women and couples actively planning a pregnancy with an indication for offering sequencing for carrier status and medically actionable secondary findings (SF) found knowledge about a particular health condition in their family (69%) as the most motivating factor for participation, tied with curiosity, and followed by reproductive planning (52%) [12]. 

### 3.4. Curiosity

In addition to altruism and personal benefit, curiosity was another motivator for participation in these studies. Interestingly, the majority of participants in the in the CPMC study (81%), the HealthSeq study (71%), and the NextGen (69%) study, all of which were conducted with unselected participants, were motivated to participate due to curiosity or general health information seeking [10,11,12]. The ClinSeq study also identified curiosity as a motivating factor, but at a much lower rate (19%) [9]. Although less often noted, or less common across studies, other motivating factors similar to curiosity included: having an interest in personal ancestry [10,11], and being adopted and wanting information about genetics [11].

## 4. Results Disclosure

In the United States, guidelines continue to recommend that disclosing genetic results in research studies, and returning SF findings be deliberated on a “protocol-by-protocol” basis [26,27]. Prior to determining whether results will be returned, serious considerations should be given to the steps that will be taken to confirm the appropriate infrastructure, support, and approval of relevant institutions (i.e., institutional review board), maintain quality in the conduct of the analysis (e.g., CLIA-certified laboratory), and incorporate participants’ preferences (for detailed guidance, see: [27]).

Analytic and clinical validity, clinical or reproductive significance, and whether a result is clinically actionable are recommended considerations for determining results disclosure. Variations in the definition of “actionable” are debated, but most often, actionable means a genetic risk can be managed through “established interventions aimed at preventing or significantly reducing morbidity and mortality” [28,29]. From a public health perspective, the advantage of providing results to participants is the anticipation that such information can be used by the person to guide individual prevention and treatment decisions. Indeed, studies indicate participants’ preference to receive results for such reasons. 

Focus group studies conducted across the United States with members of the general public (i.e., not currently participating in an LSGS study) believed results could serve as compensation for study participation, and many felt researchers had an obligation to at least offer to disclose the results, particularly in the case of treatable and preventable conditions [13,14]. Similarly, a study of a subsample of biobank participants in the OurGenes, OurHealth, OurCommunity examined preferences for hypothetical results disclosure and found most (90%) believed it was very or somewhat important to receive their results [15]. Although there was a preference to receive results, there was divergence in the type of results people wanted to receive.

### 4.1. The Type of Results People Want

Studies with members of the general public found most participants wanted to receive all possible results, including conditions for which treatment is not currently available, but most did not want to receive variant of uncertain significance (VUS) results [13,14]. Consistent with views of the general public, the vast majority of a sample of mostly healthy participants in ClinSeq (*n* = 311), who will receive results as a condition of their participation, indicated they wanted to learn all of their results (95%) including those for which no intervention is available [16]. A focus group study of healthy participants and those at risk of CAD in the ClinSeq study confirmed the preference to receive all positive results, including those not medically actionable (i.e., diseases for which there is no known treatment) [17]. 

In contrast with perceptions of the general public and those in the ClinSeq study, healthy biobank participants, who would not receive results, were more favorable about receiving results for which an intervention or treatment was available [15]. Similarly, an LSGS study of cancer patients who would receive only cancer-related results found participants were less interested in receiving all results, only 63% indicated a preference to receive all possible results, and 6% wanted only those relevant to their medical care [18].

Studies of the general public, and of LSGS participants who would not receive results, found less favorable attitudes towards receiving uncertain results (i.e., VUS) [13,14,15]. In contrast, participants in LSGS studies who would receive results were interested in receiving VUS. Most (84%) cancer patients who would receive cancer-related results preferred to receive VUS; all wanted to receive updates in the case of VUS reclassification [18], and a significant majority of participants in the ClinSeq study wanted to receive VUS [16].

One study of the general public found result accuracy and conclusiveness were not critical to participants, they understood and expected that information from research could change over time [13]. However, another study of participants in the general public and one with healthy individuals found result accuracy was important [10,14]. Specifically, participants believed the lab should be reliable, and results should be conclusive and have a known correlation with health and disease prior to being shared with the participant. 

### 4.2. What People Plan to Do with Their Results

Consistent with arguments for providing participants with results, that it may help promote preventive behavior, studies found participants planned to use results to engage in preventive health behaviors, and would share information with their family members. In addition, participants shared that genetic test results could be useful to them for other reasons, for example, to plan for the future. 

Members of the general public reported a desire to receive all SF because they believed it could improve their health by directing treatment or disease prevention and through changing their health-related behaviors [13]. Similarly, other qualitative studies uncovered participants’ perceptions of perceived benefits from learning genetic results. Cancer patients who consented to participate in an LSGS study and who would receive results wanted this information because they believed the knowledge would help with medical decision-making and prevention decisions, including altering their lifestyle [18]. Participants who would receive results as part of ClinSeq wanted to know their results for preventive reasons, including improving diet and exercise [16].

### 4.3. Participant Perspectives on Actionability 

In addition to assisting with their own medical and preventive health decisions, participants viewed actionability to be important, as in, being able to “do” something with the information. Among the general population, such actions included: informing family members of risk, making reproductive decisions, working for environmental action or remediation, life and financial planning, and participating in future research [13,14]. Such views about actionability were also found among participants in LSGS studies who were asked about hypothetical results disclosure. Cancer patients believed results could help them prepare financially and psychologically for their future [18]. A third of a cohort of healthy participants in ClinSeq wanted to know their results to inform their children and family members [16]. Participants in the ClinSeq focus group believed results would allow for “peace of mind” and “more control” in the future [17]. When considering the development of guidelines for results return, it is important to recognize the general public may hold a broader definition of “actionable,” and view results as personally useful, even in the absence of clinical intervention. 

Of note, a study which explored perceived utility as a motivation for participation in the MedSeq study identified trust as a driving factor [19]. Participants generally fell into one of three groups: enthusiasts, health conscious, and skeptics (i.e., lowest perceived utility). Enthusiasts believed results had utility beyond medical purposes, including family and end-of-life planning. Health conscious believed results only had utility for medical purposes, and skeptics did not believe results would have personal utility. Trust in result interpretation and dissemination was the only predictor of utility, skeptics reported the least trust [19]. Additional studies that identify other predictors of participant preference may be useful for determining what information participants want to receive, and in some cases, anticipating how they may use results. 

### 4.4. Concerns about Receiving Results

The preponderance of respondents across studies were favorable about receiving results, but some studies identified concerns. For example, cancer patients raised the possibility to experience negative psychosocial outcomes in response to learning about a serious untreatable disease, but they noted that their experience with a cancer diagnosis helped them to feel better able to cope with such genetic test results [18]. Often, participants’ concerns about potential negative consequences of learning results emerged after additional discussion. Focus group participants in the ClinSeq study indicated a preference to receive all results, yet upon further discussion about possible results, participants experienced hesitations, and some reported not wanting to learn about untreatable progressive disease risk [17]. By providing an extended discussion about the possible outcomes from learning about specific types of disease, participants changed their minds, raising concerns about how informed their initial preferences were. 

Collectively, study results indicate people desire to receive all genetic results for value beyond clinical prevention or treatment. The general public was not interested in results which were uncertain, but participants enrolled in studies which included result disclosure as a condition of enrollment wanted all results, including VUS. Similarly, additional discussions about what each result means in a focus group led to some participants changing their preference, which suggests participants must be provided with clear informed consent which describes possible results and the implications of those results (i.e., psychosocial implications) in detail prior to making decisions about participating and potentially receiving results. Such results highlight the importance of providing anticipatory guidance, encouraging participants to consider possible thoughts and emotions they may experience with the possible range of outcomes [30]. 

## 5. Discussion and Conclusions

The increased focus on precision medicine and the use of LSGS in research studies and clinical practice has implications for public health. White papers, perspectives and opinions of those in public health have identified the PMI’s potential and the pitfalls from recruitment to the return of results. Beginning with recruitment, we identified altruism, personal and family benefit, and general curiosity among the most common motivators for participating in LSGS studies. In all but one study [9], participants identified more than one motivating factor for participating. Those affected or at risk were slightly more likely to identify altruism as a motivating factor compared with the general population, who were most motivated by personal benefit and curiosity. Given the vast majority of participants in LSGS studies are likely to receive negative results, additional research should examine how to balance participants’ excitement about the prospect of receiving personal health information with the reality that most results will be negative. 

There is considerable debate in the research community over whether LSGS studies should return results to participants [31]. Concerns have been raised by researchers about providing participants with genetic test results due to the potential for a participant to conflate research and clinical care. Questions about appropriateness, training, and boundary blurring are raised when a researcher provides SF results to a participant [7,26,32]. How to manage the logistics of delivering the information, and what to tell participants whose phenotypes are not indicative of the condition needs to be considered [33,34]. Conversely, others feel a sense of responsibility due to the potential for the participant or patient to undergo disease prevention or early-detection behaviors [35,36,37]. The recent Consensus Study Report from the National Academy of Sciences committee on Return of Individual-Specific Research Results Generated in Research Laboratories is moving away from strict recommendations against returning results to research participants [27]. This move appears to recognize the interest of research participants in learning about their genetic health as well as a move towards better laboratory testing in research. 

In this review, we found members of the general population and participants in ClinSeq wanted to receive all genetic results, except VUS. The most common reasons for wanting results were consistent with motivations for participating and included a sense of rights or ownership, believing the information can inform future decision-making and health behavior, and the desire to share information with their family members. In contrast, studies of LSGS participants found the majority was favorable towards receiving results, but preferred results for which an intervention was available, and wanted VUS results. Given preference variability, particularly related to SF and even more so VUS, suggestions have been made to allow participants the option to make ongoing choices and modify their preferences for the type of results they receive [14]. 

A focus group study, which probed more deeply into the potential consequences of learning of certain results [17], found participants hesitated in their initial desire to receive all results. Additional research should examine participants’ understanding of genetic results, including possible implications to their psychosocial well-being, and how their understanding (or lack thereof) informs their preferences. Determining key information and identifying appropriate levels of understanding could be useful to guiding informed consent processes for research and clinical practice. Allowing participants to select which results they will receive in absence of complete understanding of the consequences could cause negative psychosocial outcomes and impact future recruitment efforts.

Studies in this review examined hypothetical preferences, or were conducted on participants who agreed to participate in a study where genomic sequencing information will be returned to them in the future. Those not enrolled in an LSGS study and those unaffected individuals in the process of enrolling in a study where results will be returned were more likely to be favorable towards receiving results compared with those who were affected or who enrolled in a study where results would not be returned. Given these differences, research which allows participants a choice about receiving results could improve participant heterogeneity. Additionally, studies where participants were told they would not receive results at enrollment (e.g., some biobank studies), but are now considering returning results, should do so with caution. Research conducted on the process of actually returning results in such cases could inform our understanding of actual preferences. 

Most studies in this review included participants who could be classified as “early adopters of new technologies”, specifically those who are white, of higher socioeconomic status, higher education, and more interested in taking risks [9]. As such, extrapolating their intentions and experiences with LSGS to the general population is not reasonable. Although this information provides a foundation for understanding public perceptions, it is unlikely that it will be consistent with the general population [38]. A conclusion reached by nearly every study was the need for additional research on diverse populations in terms of location (urban vs. rural), accessibility, ethnicity, race, education, socioeconomic status and drawing participants who have varied experience with disease, illness, and knowledge or experience with genetics [18]. 

Strategies to engage minority and underserved communities include making initial contact by phone, using community-based strategies such as engaging community leaders, and involving community members with research-based activities throughout the duration of the study [39]. Continued efforts to identify best practices for recruitment of minority and underserved populations is urgent for behavioral and biomedical reasons. Genetic heterogeneity exists between population groups, which impacts disease risk (e.g., some diseases are more common than others in certain populations), and treatment responses [40]. Failing to include minority racial and ethnic populations in LSGS studies will increase knowledge of disease risk in majority populations only, thereby perpetuating health disparities [41]. Continued research to determine methods for improving trust of racial/ethnic minority populations and improving recruitment to LSGS studies is a priority. 

LSGS studies are beginning to return results to their participants, and data soon will be available about the type of actions participants take in response to learning about their genetic risk. Despite the desire to receive results to inform choices about health protective behaviors, little is known about how participants will use this information. A revised and updated Cochrane review published in 2016 found the disclosure of genetic risk has little or no effect on health-related behavior [42]. These results are not surprising. For decades, health behaviorists have known information alone does not change behavior. The development of health behavior theories for communicating risk information such as the Health Belief Model [43], the Extended Parallel Process Model [44], the Transtheoretical Model [45] were the direct result of this acknowledgement. Information delivered in absence of a theoretical framework is unlikely to be motivating. Furthermore, behavior change is difficult. Even among patients who experienced life-changing health events such as a heart attack or cancer often fail to sustain behavior change over time. 

In addition to asking about motivations to participate in the aforementioned CPMC study, Gollust, Gordon, Zayac, Griffin, Christman, Pyeritz, Wawak and Bernhardt [11] also asked about participants’ intentions to share their results with their healthcare providers. Perhaps consistent with the desire for genetic information to improve their health, the vast majority (91.7%) stated they were likely or very likely to share their results with their physicians. In part, the researchers noted that participants in the CPMC study were encouraged to share their results with their healthcare providers when they enrolled in the study, which may have skewed these results. Identifying pathways between genetics professionals (who disclose the result) and healthcare providers who have ongoing contact and can monitor and encourage patients’ prevention behavior, may have advantages for adherence. Although actual follow-up with physicians was low in the CPMC study, the authors indicated that primary care physicians may not have the training required to assist patients in interpreting and managing their healthcare based on genetic test results. They suggested additional research which follows early adopter’s use of primary care services after receiving genetic test results to evaluate whether regulatory guidance is needed on a public health level [11].

The ability for LSGS studies and the “All of Us” study to positively impact public health will largely be determined by several important factors that will require additional investigation. First, continued efforts are needed to identify effective methods for recruiting a diverse cohort of participants. Second, additional research is needed to determine which results will be most likely to improve prevention behaviors and the least likely to cause negative psychosocial consequences. Preferences related to race/ethnicity, gender, age, and other socioeconomic factors should continue to be explored. Finally, determining communication strategies to disclose results which have the greatest potential to motivate prevention behaviors, and identifying pathways to improve adherence will be critical. The inclusion of a diverse cohort, development of guidelines for results disclosure, and messages and systems to support prevention behavior will lay the groundwork to achieve the promise of precision public health. 

## Figures and Tables

**Table 1:** Included Studies.

In Text Citation	Article	LSGS	Methods	Purpose	Population	Disease	Return of Results
[9]	Facio, F.M., Brooks, S., Loewenstein, J., Green, S., Biesecker, L.G., & Biesecker, B.B. (2011). Motivators for participation in a whole-genome sequencing study: Implications for translational genomics research.	ClinSeq	Quantitative-participants completed surveys	To understand motivations and expectations of individuals who are choosing to have their whole genome or exome sequenced, and have the opportunity to learn about their results in the future	Individuals age 45–65 with a risk of developing coronary artery disease, including asymptomatic and symptomatic individuals	Individuals age 45–65 with a risk of developing coronary artery disease, including asymptomatic and symptomatic individuals	Yes; Selected, but not all- results are returned; a subset of data considered high-throughput results was determined as appropriate to return
[10]	Sanderson, S.C., Linderman, M.D., Suckiel, S.A., Diaz, G.A., Zinberg, R.E., Ferryman, K., Wasserstein, M., Kasarskis, A., & Schadt, E.E. (2016). Motivations, concerns and preferences of personal genome sequencing research participants: Baseline findings from the HealthSeq project.	HealthSeq	Mixed methods-participants completed questionnaires and in-depth interviews at multiple points during study	To assess motivations and concerns that participants have about genome sequencing; to understand preferences around return of results and informed consent	Unselected healthy adult population	Various diseases	Yes; A range of results including Alzheimer’s, type 3 diabetes, rare disease-associated variants, ancestry, and pharmacogenomics; participants also offered raw data
[11]	Gollust, S.E., Gordon, E.S., Zayac, C., Griffin, G., Christman, M.F., Pyeritz, R.E., Wawak, L., & Bernhardt, B.A. (2012). Motivations and perceptions of early adopters of personalized genomics: Perspectives from research participants.	Coriell Personalized Medicine Collaborative	Quantitative-participants completed surveys	To ascertain motivation for enrolling, perceptions around risks and benefits, and intention to share results	Selected healthy adult populations	Various diseases	Yes; Actionable genetic variant results, non-genetic risk factors, and drug responses; participants decide whether they wish to view each actionable result
[12]	Kauffman, T.L., Irving, S.A., Leo, M.C., Gilmore, M.J., Himes, P., McMullen, C.K., Morris, E., Schneider, J., Wilfond, B.S., & Goddard, K.A.B. (2017). The NextGen study: Patient motivation for participation in genome sequencing for carrier status.	NextGen	Qualitative-participants were asked two open-ended questions about motivation to participate during an informed consent and education session with genetic counselor	To explore motivations in healthy pre-conception women to participate in genome sequencing research	Pre-conception women who were planning for pregnancy, and had undergone carrier screening	Various diseases	Yes; Medically actionable secondary findings, carrier findings
[13]	Bollinger, J.M., Joan, S., Dvoskin, R., & Kaufman, D. (2012). Public preferences regarding the return of individual genetic research results: Findings from a qualitative focus group study.	N/A-hypothetical	Qualitative-participants were divided into 10 focus groups	To explore preferences around the return of individual research results in genetic research	Unselected healthy adult population	N/A-hypothetical	N/A-hypothetical
[14]	Murphy, J., Scott, J., Kaufman, D., Geller, G., Leroy, L., & Hudson, K. (2008). Public expectations for return of results from large-cohort genetic research.	N/A	Qualitative-participants were divided into 6 focus groups	To learn values and perspectives about the return of individual research results in genetic studies	Unselected adults	Various diseases	Yes; Individual research results
[15]	Allen, N.L. Karlson, E.W., Malspeis, S., Lu, B., Seidman, C.E., & Lehmann, L.S. (2014). Biobank participants’ preferences for disclosure of genetic research results: Perspectives from the OurGenes, OurHealth, OurCommunity project.	Our Genes, Our Health, Our Community	Quantitative-participants completed a survey	To understand biobank participants preferences in the disclosure of results	Selected healthy adult patients	Various diseases	Yes; Hypothetically all results including results indicating high penetrance and risk for serious conditions
[16]	Facio, F.M., Eidem, H., Fisher, T., Brooks, S., Linn, A., Kaphingst, K.A., Biesecker, L.G., & Biesecker, B.B. (2013). Intentions to receive individual results from whole-genome sequencing among participants in the ClinSeq study.	ClinSeq	Mixed methods-participants completed surveys with open-ended questions	To learn general preferences and attitudes towards learning different types of genetic test results	Individuals age 45–65 with a risk of developing coronary artery disease, including asymptomatic and symptomatic individuals	Coronary artery disease	All results ranging from medically actionable to unknown significance
[17]	Wright, M.F., Lewis, K.L., Fisher, T.C., Hooker, G.W., Emanuel, T.E., Biesecker, L.G., & Biesecker, B.B. (2014). Preferences for results delivery from exome sequencing/genome sequencing.	ClinSeq	Qualitative-participants were divided into 6 focus groups	To understand enthusiasm towards and implications for returning genetic test results	Individuals age 45–65 with a risk of developing coronary artery disease, including asymptomatic and symptomatic individuals	Coronary artery disease	All results ranging from medically actionable to unknown significance
[18]	Hitch, K., Joseph, G., Guiltinan, J., Kianmahd, J., Youngbolm, J., & Blanco, A. (2014). Lynch Syndrome Patients’ Views of and Preferences for Return of Results Following Whole Exome Sequencing.	N/A	Qualitative-participants completed individual interviews	To explore preferences of cancer patients about return of results from WES	Patients previously diagnosed with Lynch syndrome but received uninformative negative Lynch syndrome genetic results through traditional genetic testing	Lynch Syndrome	Yes; They only would receive cancer-related results generated from WES
[19]	Lupo, P.J., Robinson, J.O., Diamond, P.M., Jamal, L., Danysh, H.E., Blumenthal-Barby, J., Lehmann, L.S., Vassy, J.L., Christensen, K.D., & Green, R.C. (2016). Patients’ perceived utility of whole-genome sequencing for their healthcare: Findings from the MedSeq project.	MedSeq	Quantitative-participants completed surveys	To understand participants’ perceived utility, and how attitudes, behaviors, and demographic factors predict perceived utility	Healthy primary care participants 40–70 years old; Cardiology patients >18 years old	Various diseases in healthy participants; cardiovascular disease (hypertrophic and dilated cardiomyopathy) in cardiology patients	Yes; All results, including results in which clinical significance is uncertain

Note: LSGS: Large-scale genomic sequencing study; N/A: Not applicable.
